# Yield of Protein Crystallization from Metastable Liquid–Liquid Phase Separation

**DOI:** 10.3390/molecules30112371

**Published:** 2025-05-29

**Authors:** Shamberia Thomas, Joel A. Dougay, Onofrio Annunziata

**Affiliations:** Department of Chemistry and Biochemistry, Texas Christian University, Fort Worth, TX 76109, USA; shamberia.thomas@tcu.edu (S.T.); joel.dougay@tcu.edu (J.A.D.)

**Keywords:** NaCl, HEPES, LLPS

## Abstract

Preparative protein crystallization is regarded as an economically sustainable protein purification alternative to chromatography in biotechnological downstream processing. However, protein crystallization is a not-well-understood process that is usually slow and poorly reproducible. A promising strategy for enhancing protein crystallization is exploiting the metastable liquid–liquid phase separation (LLPS) of protein solutions. Here, we report an enhancement of lysozyme-crystallization yield by using a combination of two additives under LLPS conditions. The first additive, NaCl (0.15 M), is necessary to introduce protein–protein attractive interactions and induce LLPS by lowering temperature. The second additive, 4-(2-hydroxyethyl)-1-piperazineethanesulfonate (HEPES, 0.10 M, pH 7.4), accumulates in the metastable protein-rich liquid phase and thermodynamically stabilizes lysozyme crystals. We found that this combination of additives leads to crystallization yields of higher than 90% under LLPS conditions at a lysozyme concentration of 5% by weight and a fairly low ionic strength (0.2 M) within an operational time of the order of one hour. This crystallization yield is more than three-fold larger than that obtained from samples containing NaCl without HEPES at the same pH and ionic strength. Moreover, we determined crystallization yield as a function of incubation time, and temperature below and above the LLPS boundary. As crystallization temperature intersects with LLPS temperature, a significant increase in crystallization yield is observed. This is consistent with LLPS boosting protein crystallization. Our work suggests a possible strategy for increasing the crystallization success of other proteins, with applications in protein purification.

## 1. Introduction

In recent years, the high demand for protein in pharmaceutical and biotechnological fields has resulted in an increase in the manufacturing of proteins and their applications as drugs and catalysts. The advances in cell culture technology in upstream processing can currently produce large quantities of protein. This has shifted the bottleneck of protein production towards protein purification in downstream processes [1,2]. Although chromatography is very reliable, it is also expensive and cannot be easily applied to large amounts of protein. These shortcomings have increased the need for more economically sustainable alternatives to chromatography for protein purification [3,4,5]. One promising alternative is preparative protein crystallization [4,6,7,8,9]. Protein crystallization, even when producing small microcrystals, can be the ultimate purification step since it relies on the stringent structural requirements for protein crystal formation to exclude impurities from the lattice of the three-dimensional ordered protein array. However, protein crystallization is a complex, poorly reproducible, and not-well-understood process [10,11,12,13,14,15]. Thus, extending crystallization to many other proteins to achieve their purification remains a very difficult task. Indeed, its implementation typically requires extensive crystallization screening [14,16].

It has been previously proposed that the metastable [17,18,19] liquid–liquid phase separation (LLPS) of protein solutions [20,21] represents a valid strategy favoring protein crystallization [22,23,24,25,26]. A central feature of LLPS is the formation of protein-rich micro-droplets [20]. Due to LLPS’ metastability, protein-rich droplets represent intermediate states for the nucleation of protein crystals. One explanation supporting the idea that protein-rich droplets enhance protein nucleation is that the protein-rich liquid layer surrounding the crystal nucleus lowers its interfacial energy (wetting mechanism) [22]. Another explanation is that crystal nucleation must inherently start from small-protein liquid-like clusters that then evolve into ordered crystalline nuclei (two-step mechanism) [15,27].

LLPS, which is typically induced by cooling aqueous protein samples, can be thermodynamically described by considering a temperature–concentration phase diagram showing the LLPS phase boundary together with the protein–crystal solubility curve [17,18,19,28]. Both phase transitions are driven by solvent-mediated protein–protein attractive interactions in the liquid state [29]. LLPS is metastable with respect to protein crystallization but can be observed because protein crystallization is a relatively slow process. LLPS metastability has been linked to the short-term nature of protein–protein interactions [17,29].

**Figure 1:**
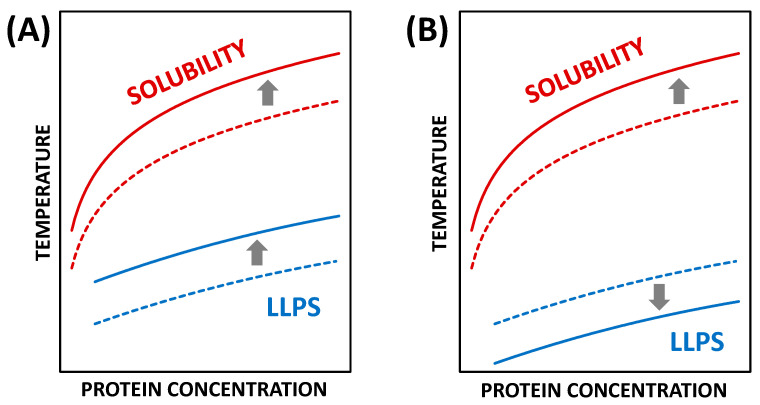
(**A**) Schematic phase diagram with temperature and protein concentration on the *x*- and *y*-axes, respectively. This qualitative phase diagram shows solubility and LLPS boundaries. The effect of an additive on the phase diagram is described by shifts in the phase boundaries. An increase in the concentration of a traditional salting-out agent produces an upward shift in the two phase boundaries (from dashed to solid curves). Examples of actual phase diagrams showing the effect of NaCl concentration on phase boundaries of HEWL solutions are reported in References [23,32]. (**B**) A schematic phase diagram qualitatively showing that the addition of HEPES produces an upward shift in the HEWL solubility boundary (salting-out) but a downward shift in the LLPS boundary (salting-in). The experimental phase diagram describing how the addition of 0.10 M HEPES impacts phase boundaries of HEWL solutions is available in Reference [38].

The location of LLPS and the solubility boundaries in the phase diagram depends on ionic strength, pH, and the concentration and chemical nature of additives [23,30,31,32]. The effect of an additive capable of increasing protein–protein attraction energy and promoting phase separation is schematized in Figure 1A. This diagram first shows the LLPS and solubility phase boundaries in the absence of the target additive (dashed curves). The phase domain below the solubility curve describes supersaturated states where the protein concentration is higher than the crystal solubility. The LLPS boundary is metastable because it is located within this supersaturated region. Protein solutions at temperatures below the LLPS boundary will quickly become cloudy due to the formation of coexisting protein-rich and protein-poor liquid phases [33]. These liquid phases may eventually generate protein crystals. An additive that increases protein–protein attraction reduces the thermodynamic stability of homogenous protein samples. This causes both the solubility and the LLPS boundary to shift toward higher temperatures (see solid curves in Figure 1A). This type of additive is denoted as a salting-out agent because it favors protein precipitation. In contrast, an additive that shifts the solubility and LLPS boundary toward lower temperatures is denoted as a salting-in agent. In other words, it acts as a protein solubilizer. It is important to note that, in this paper, we use the terms salting-in and salting-out to specifically describe the observed effect of an additive on a given phase boundary. Thus, an additive could be a salting-in agent with respect to a phase transition and a salting-out agent with respect to another phase transition.

Hen-egg-white lysozyme (HEWL) is a model protein for crystallization studies, with NaCl being the most successful crystalizing agent for this protein [34,35,36]. The effect of NaCl on lysozyme solution is schematized in the phase diagram of Figure 1A. Interestingly, our previous work [37,38] has shown that the addition of 4-(2-hydroxyethyl)-1-piperazineethanesulfonate (HEPES) to lysozyme-NaCl solutions produces a more complex effect on the phase diagram. As shown in Figure 1B, HEPES moves the solubility curve toward higher temperatures while shifting the LLPS boundary toward lower temperatures [38]. In this system, HEPES acts as a salting-out agent for crystallization but as a salting-in agent for LLPS. The salting-in effect of HEPES on LLPS was linked to HEPES preferential binding to HEWL [37,39]. This was demonstrated by showing that HEPES accumulates in the protein-rich phase [37]. However, HEPES preferential binding to protein should also enhance protein crystals solubility [39], in disagreement with the experimental results. The unexpected salting-out effect of HEPES on HEWL crystallization is explained by the hypothesis that HEPES promotes physical cross-linking interactions between neighboring protein units in the crystal lattice. This hypothesis aligns with the general belief that multi-functional organic molecules are beneficial in protein crystallography [40].

**Figure 2:**
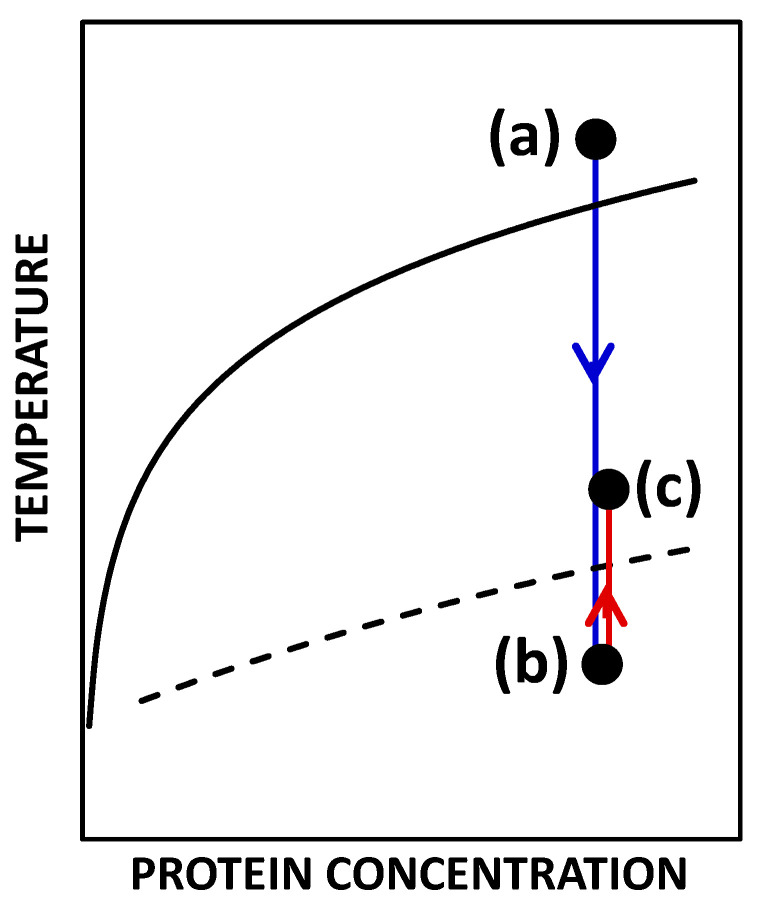
Schematic temperature–concentration phase diagram qualitatively showing solubility (solid curve) and LLPS boundaries (dashed curve). Point (a) indicates a protein solution state that is thermodynamically stable with respect to crystallization and LLPS. In this work, the coordinates of point (a) are typically a HEWL concentration of 50 g·L^−1^ and temperature of 50 °C. At this protein concentration, the solubility and LLPS temperatures are 38 °C [37] and −13.6 °C, respectively. The temperature of the protein solution is then quickly lowered until it reaches the state indicated by point (b). In this work, the temperature of point (b) is typically −15.0 °C. Here, LLPS occurs and should promote the nucleation of protein crystals. After a well-defined incubation time, the sample temperature is then raised above the LLPS temperature until it reaches the state indicated by point (c). Here, crystal growth should further increase crystallization yield.

Interestingly, after inducing LLPS by cooling, aqueous samples of HEWL containing both NaCl (0.15 M) and HEPES (0.10 M) are observed to rapidly generate a copious amount of compact tetragonal microcrystals, even at fairly low ionic strength of 0.2 M [37]. This outcome is particularly appealing for protein purification. Indeed, as shown in Figure 2, it is possible to devise a protein crystallization protocol in which a homogeneous protein solution is first quenched to a temperature below the LLPS temperature to obtain LLPS and enhance crystal nucleation. After a well-defined incubation time, the sample temperature is then raised above the LLPS temperature to dissolve the protein-rich liquid phase. This favors the growth of protein crystals and further increases crystallization yield. These crystals could then be separated from the aqueous media and further purified from small ions and molecules by employing standard dialysis techniques.

Remarkably, aqueous samples of HEWL without HEPES but with extra NaCl (0.18 M, to match the ionic strength) exhibit a significantly lower crystallization output during visual inspection. This result is noteworthy because NaCl is regarded as the most successful crystallizing agent and salting-out additive for HEWL [41]. Moreover, HEPES, which is a member of the Good’s buffer family, is often believed to have a negligible impact on biochemical systems [42,43]. Our previous results instead showed that HEPES significantly impacted the crystallization of HEWL [37,38].

From these results, it is possible to hypothesize that the success rate of LLPS-based protein crystallization protocols can be increased if two additives are combined. One additive is a traditional salting-out agent, which is necessary to induce LLPS and establish attractive protein–protein interactions. The second additive, which should be a water-soluble multi-functional organic molecule exhibiting weak ligand properties, should modify phase-diagram topology by increasing the metastability gap between the LLPS boundary and crystal solubility curve. The organic molecule should also accumulate in the protein-rich liquid phase and ultimately act as a physical crosslinker inside protein crystals.

Although the effect of HEPES on HEWL crystallization was previously described based on turbidity profiles [37,38], quantitative data on the yield of protein crystallization are missing. This information is particularly valuable for examining how crystallization output depends on sample incubation time and crystallization temperature that are below and above the LLPS temperature. Thus, in this paper, we report the experimental crystallization of the yield of HEWL crystallization in the presence of HEPES.

It is important to note that crystal nucleation is an inherently stochastic process. This means that crystallization outcome may vary significantly, even among identical samples. This may lead to poor reproducibility for the experimental yield data. However, since HEWL crystallization with HEPES is fairly fast, and leads to a large number of protein crystals, many of the values of the crystallization yields reported in this work have a good reproducibility, with most variations in the order of 5% or less. This means that the dependence of crystallization yield on crystallization temperature can be used to quantify the role of LLPS in the protein crystallization rate.

## 2. Results and Discussion

To examine the role of HEPES in the LLPS-mediated crystallization of HEWL, we determined the crystallization yield, *Y*, for several samples with a HEWL concentration of 50 g·L^−1^. The quenching temperature was *T*_Q_ = −15 °C, and the incubation time at this temperature was Δ*t*_Q_ = 30 min. To clear away LLPS, the sample temperature was then raised to 2 °C above the LLPS temperature. Here, samples were incubated for an additional 30 min. We focused on two aqueous systems of this protein. Both systems share the same pH (7.4) and ionic strength, *I* = 0.20 M, to match the electrostatic screening conditions. These two systems differ in the composition of their additives, with one containing both HEPES and NaCl (0.15 M NaCl with 0.10 M HEPES buffer) and the other containing NaCl only (0.183 M NaCl, with a small amount of 20 mM Tris buffer). Note that both mixtures can slowly generate protein crystals. This was verified after overnight sample incubations at 2 °C or 22 °C.

Our results are reported in Table 1. Here, we can appreciate that LLPS temperature decreases in the presence of HEPES. As previously mentioned, HEPES acts as a salting-in agent because it suppresses LLPS (see Figure 1B). Thus, NaCl is necessary to induce LLPS. Consistent with this, HEWL solutions that contain HEPES but not NaCl do not exhibit LLPS.

Our experiments reproducibly show that HEPES dramatically increases crystallization yield, consistently reaching values superior to 90% (see Table 1). Specifically, the addition of HEPES to NaCl at a constant ionic strength produces more than a three-fold increase in crystallization yield. To assess how close the crystallization yield is to its theoretical limit, we consider HEWL solubility at 2 °C. Its value is 3.2 g·L^−1^ in HEPES-NaCl and 6.5 g·L^−1^ in the HEPES-free NaCl systems [38]. To calculate maximum theoretical yields at this temperature, we can use mass conservation to write *Y* = 1 − (*C_f_*/*C_i_*)·(*V_f_*/*V_i_*), where *C_i_* and *C_f_* are the initial (50 g·L^−1^) and final (solubility) concentration, and *V_i_* and *V_f_* are the corresponding solution-phase volumes, with *V_f_*/*V_i_* ≈ 1. To precisely characterize the volume ratio, we write *V_f_*/*V_i_* = 1 − *V*_C_/*V_i_*, where *V*_C_ is the volume of the crystalline phase. This can be written as *V*_C_ = (*C_i_*·*V_i_* − *C_f_*·*V_f_*)/*ρ*_C,_ where *ρ*_C_ is crystal density. We finally obtain this expression of yield: *Y* = 1 − (*C_f_*/*C_i_*)·(*ρ*_C_ − *C_i_*)/(*ρ*_C_ − *C_f_*). If we use *ρ*_C_ = 1.24 g·cm^−3^ for tetragonal HEWL [44], we can calculate the maximum yields of 94% and 88% in the HEPES-NaCl and HEPES-free NaCl systems, respectively. We therefore conclude that the yield of HEWL crystallization in the presence of HEPES has virtually reached its theoretical limit.

Since HEPES is a buffer, we also explored the effect of another commonly employed buffer on crystallization yield. We specifically chose the phosphate buffer not only due to its application at an approximately neutral pH but also for its known ability to induce the LLPS of HEWL solutions [21] even in the absence of NaCl (see LLPS temperatures in Table 1). Systems containing a phosphate buffer (0.10 M) and NaCl (0.10 M), or just a phosphate buffer at a higher concentration (0.20 M), exhibit low crystallization yields (6%) even if their ionic strengths are appreciably higher than that used in the HEPES case. We therefore conclude that the ability of a phosphate buffer to produce HEWL crystals is relatively poor, even if it can be successfully used to induce LLPS. It is, however, interesting to note that the substitution of NaCl (0.10 M) with HEPES (0.10 M) in the phosphate buffer again produces a dramatic increase in crystallization yield.

**Figure 3:**
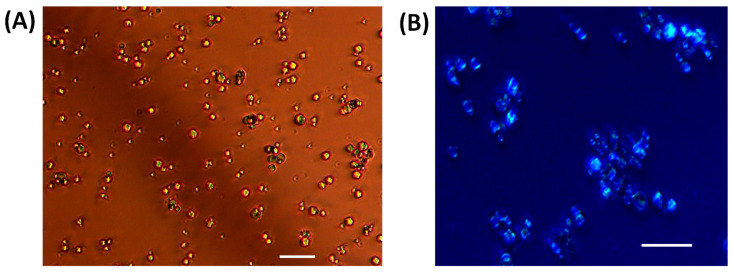
(**A**) Direct field image of HEWL microcrystals. (**B**) Polarized image showing the birefringence of HEWL microcrystals. Horizontal bars are 20 μm.

We believe that the high yield (>90%) of the LLPS-mediated crystallization of HEWL with the combination of NaCl and HEPES provides an optimal strategy for protein purification. Indeed, this is achieved by cooling samples with a fairly low concentration of protein (≈5% by weight) and additives (0.15 M NaCl + 0.10 M HEPES) within an operational time that is of the order of one hour. In Figure 3, direct-field and polarized microscopy images of the HEWL crystals generated from this system are shown. These crystals exhibit a size of about 1–2 μm. While these microcrystals may not be used for the determination of protein structure using traditional X-ray crystallography [11], they can be useful for applications in the purification and separation of proteins. Furthermore, this type of protein microcrystal may be still suitable for the determination of protein structure using emerging techniques such as femtosecond crystallography using X-ray free-electron lasers (XEFL) [45] and Microcrystal Electron Diffraction in cryo-electron microscopy [46].

**Figure 4:**
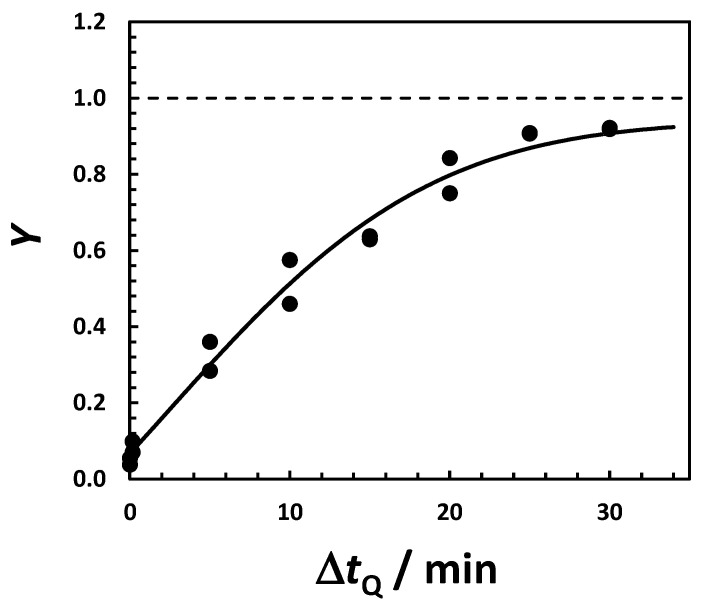
Yield of HEWL crystallization, *Y*, in 0.10 M HEPES (pH 7.4) and 0.15 M NaCl as a function of quenching time, Δ*t*_Q_, at HEWL concentration 50 g·L^−1^ and quenching temperature *T*_Q_ = −15 °C. All samples were then incubated at 2 °C for 30 min. Solid curve is the fit through the data based on the exponential function *Y* = *Y*_0_ + (0.94 − *Y*_0_)·[1 − exp(−*j*·Δ*t*_Q_)], where *Y*_0_ = 0.07 is the initial yield of crystallization, 0.94 represents the maximum theoretical yield based on crystal solubility, and *j* = 0.052 + 0.0019· Δ*t*_Q_ may be described as the apparent rate of crystal nucleation. Horizontal dashed line indicates *Y* = 1.

The high yield of HEWL crystallization observed in the NaCl-HEPES system is reproducible and measurable within a relatively short time. This means that we can use measurements of crystallization yields to investigate the effect of time and temperature on crystallization rate. We start by examining the effect of the incubation time, Δ*t*_Q_, on the yield of protein crystallization at a constant quenching temperature (*T*_Q_ = −15 °C) and HEWL concentration (50 g·L^−1^). In all cases, heterogeneous mixtures were subsequently incubated at 2 °C above the LLPS temperature for 30 min. In Figure 4, it is shown that crystallization yield, *Y*, increases with Δ*t*_Q_. Its value is 10% when Δ*t*_Q_ = 10 s and becomes ≈80% when Δ*t*_Q_ = 20 min. It then plateaus as yield becomes higher; ≈90% is near its theoretical maximum. These results show that Δ*t*_Q_ has a significant impact on crystallization yield. We attribute this effect to an increase in the concentration of protein-crystal nuclei with Δ*t*_Q_. To determine the effect of incubation time at 2 °C, experiments were also carried out with Δ*t*_Q_ fixed at 30 min. It was found that the yield was already ≈50% with only 10 s at 2 °C, before reaching values above 90% after 30 min. This additional increase in crystallization yield can mostly be attributed to protein crystal growth.

**Figure 5:**
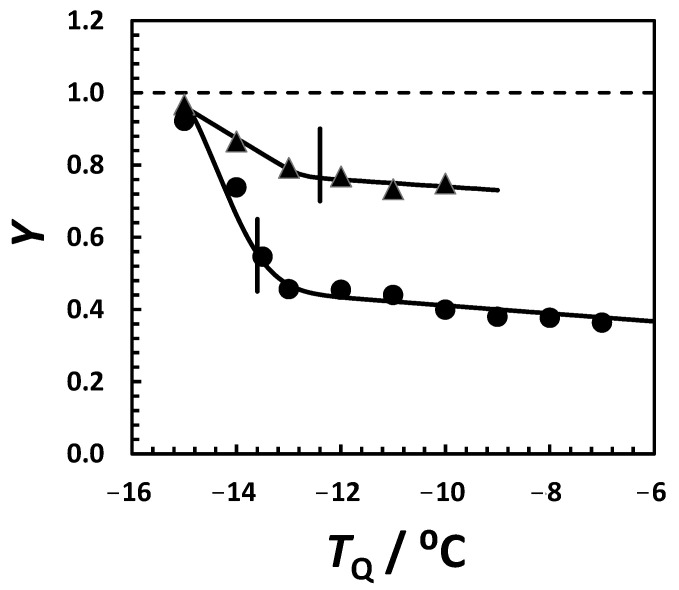
Yield of HEWL crystallization, *Y*, in 0.10 M HEPES (pH 7.4) and 0.15 M NaCl as a function of quenching temperature, *T*_Q_, at quenching time, Δ*t*_Q_ = 30 min. All samples were then incubated at 2 °C for 30 min. HEWL concentration was 50 g·L^−1^ (circles) and 80 g·L^−1^ (triangles). Vertical lines indicate LLPS temperature. Solid curves are a guide for the eye.

We also examined the effect of quenching temperature, *T*_Q_, on crystallization yield at the constant quenching time of Δ*t*_Q_ 30 min and HEWL concentration of 50 g·L^−1^. Values of *T*_Q_ were increased from −15 °C below *T*_ph_ (−13.6 °C; see Table 1) to −7 °C above *T*_ph_. As shown in Figure 5 (solid circles), crystallization yield decreases as *T*_Q_ increases. Interestingly, a significant change in the slope of *Y*(*T*_Q_) is observed at *T*_Q_ ≈ −13 °C near the LLPS temperature, where *Y* ≈ 50%. We extended these measurements to protein samples at a HEWL concentration of 80 g·L^−1^, for which *T*_ph_ = −12.4 C and yields are relatively higher. Again, a significant change in the slope of *Y*(*T*_Q_) is observed in the proximity of the LLPS temperature.

This noteworthy result shows that crystallization yield significantly increases as quenching temperature decreases below the LLPS temperature. Thus, the formation of a metastable protein-rich liquid phase enhances protein crystallization. This is consistent with the ability of the protein-rich phase to lower crystal surface tension, thereby enhancing the protein nucleation rate [22]. It is important to observe that crystallization yield also remains high (≈40%) at *T*_Q_ values just above *T*_ph_. This, of course, can be attributed to the intrinsic salting-out ability of HEPES to promote HEWL crystallization. It is, however, important to note that the formation of liquid-like protein nanoclusters can occur even above *T*_ph_, in the proximity of the LLPS boundary [47]. This clustering can also promote the nucleation of protein crystals. In support of this hypothesis, theoretical studies have shown that liquid-like protein clusters are precursors for the formation of crystal nuclei (two-step mechanism for crystal nucleation) [15,27]. Thus, high crystallization yields at *T*_Q_ values just above the LLPS temperature may be related to protein clustering associated with the LLPS phenomenon.

The reproducibility of protein crystallization yields is high (variations of ≈5%) at quenching temperatures below the LLPS temperature and just above the LLPS temperature. However, it significantly reduces as quenching temperature further increases beyond the LLPS boundary. Specifically, crystallization yields above *T*_Q_ ≈ −6 °C were found to significantly vary, ranging from *Y* ≈ 5% to ≈40%. Note that large fluctuations in yield values are generally expected as crystal nucleation is a stochastic process [48,49]. Thus, our results indicate that LLPS improves the reproducibility of crystallization yields, which is a desirable feature of protein purification protocols.

**Figure 6:**
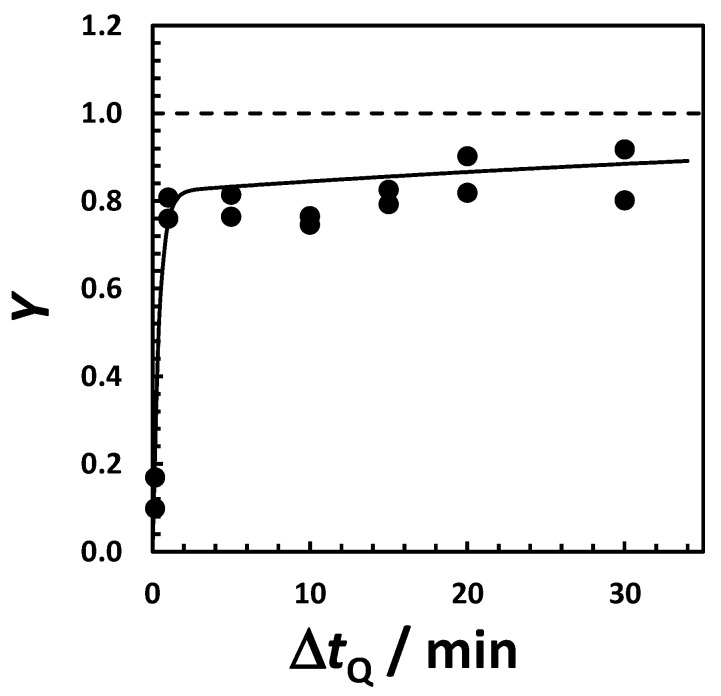
Yield of HEWL crystallization, *Y*, in 0.10 M HEPES (pH 7.4) and 0.30 M NaCl as a function of quenching time, Δ*t*_Q_, at HEWL concentration 50 g·L^−1^ and quenching temperature *T*_Q_ = −9 °C. All samples were then incubated at 2 °C for 30 min. Solid curve is a guide for the eye.

To examine crystallization yields at relatively high temperatures in the presence of LLPS, we increased NaCl concentration. Specifically, we determined that the LLPS temperature of HEWL samples at 50 g·L^−1^ in the presence of 0.10 M HEPES increased from −13.6 °C to −4.4 °C as NaCl concentration increased from 0.15 M to 0.30 M. We generally found very high yield values when NaCl concentration was 0.30 M. As illustrated in Figure 6, the crystallization yield measured at *T*_Q_ = −9 °C is ≈10% when Δ*t*_Q_ = 10 s, and reaches ≈80% when Δ*t*_Q_ = 1 min. Thus, increasing NaCl concentration in the presence of HEPES further boosts HEWL crystallization output.

**Figure 7:**
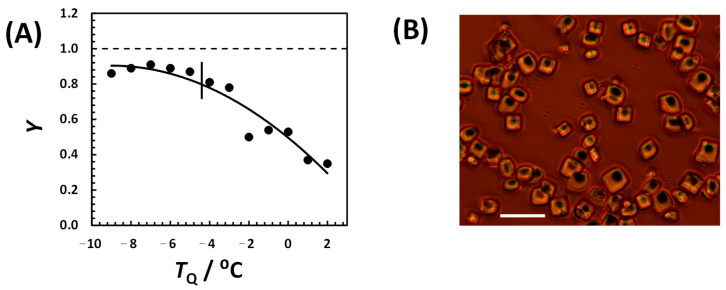
(**A**) Yield of HEWL crystallization, *Y*, in 0.10 M HEPES (pH 7.4) and 0.30 M NaCl as a function of quenching temperature, *T*_Q_, at quenching time Δ*t*_Q_ = 30 min. All samples were then incubated at 2 °C for 30 min. HEWL concentration was 50 g·L^−1^. Vertical line indicates LLPS temperature. Solid curve is a guide for the eye. (**B**) Direct field image of HEWL microcrystals. Horizontal bar is 20 μm.

As in the previous experiments with 0.15 M NaCl, we carried out measurements of crystallization yield as a function of *T*_Q_ at Δ*t*_Q_ = 30 min. Our results are shown in Figure 7A. At the higher NaCl concentration, crystallization yield also increased as quenching temperature decreased. However, high values of Y ≈ 90% were already reached near the LLPS temperature. Thus, the change in the slope of *Y*(*T*_Q_) to below *T*_ph_ could not be observed in these conditions. 

Increasing the NaCl concentration increases not only crystallization yield but also the size of HEWL microcrystals. This is illustrated in Figure 7B. From the geometric shape of these crystals, we can appreciate some of the crystalline traits characteristic of HEWL’s tetragonal morphology [35,37,50].

## 3. Materials and Methods

***Materials:*** Two-times recrystallized and lyophilized Hen-Egg-White-Lysozyme (HEWL) was purchased from Worthington Biochemical Corporation, Lakewood, NJ, USA and used without further purification. Analytical reagent grade 4-(2-hydroxyethyl)-1-piperazineethanesulfonic acid (HEPES, 238.30 g∙mol^−1^, Sigma Aldrich, St. Louis, MO, USA), Tris (hydroxymethyl) amino methane, (Tris, 121.14 g∙mol^−1^, Fischer Sci., Hampton, NJ, USA), sodium chloride (NaCl, Chem-Impex, Wood Dale, IL, USA), sodium hydroxide (NaOH, 40.00 g∙mol^−1^, Fischer Sci., MA, USA), hydrochloric acid (HCl 36.5–38.0% *w*/*w*, Pharmca, Hydro, OK, USA), sodium phosphate monobasic (NaH_2_PO_4_∙H_2_O, 137.99 g∙mol^−1^, Fischer Sci., NJ, USA) and sodium phosphate dibasic (Na_2_HPO_4_, 141.96 g∙mol^−1^, Fischer Sci., NJ, USA) were used without further purification. Deionized water was passed through a four-st, age Millipore filter system to provide high-purity water for all the experiments.

***Sample Preparation:*** Protein-free aqueous media were prepared by weight in volumetric flasks. All samples were prepared at pH 7.4. The targeted value of pH 7.4 was obtained by adding small amounts of aqueous ≈ 0.1 M NaOH or ≈0.1 M-HCl prior to final-volume adjustments. A HEWL aqueous solution in a given aqueous medium was prepared by first mixing protein powder with the appropriate NaCl-free buffered solutions, followed by dialysis (Amicon, Millipore, Burlington, MA, USA, 10 kDa cutoff membrane) against the same stock solution. The resulting HEWL solution was then transferred into a centrifugal filter device (Amicon Ultra, Millipore, 10 kDa cutoff membrane) where it was completely dialyzed into the desired aqueous medium to the final targeted protein concentration (e.g., 50 g∙L^−1^) using a centrifuge (Allegra 25R centrifuge, Beckman Coulter, Brea, CA, USA). If needed, the dilution of these concentrated protein solutions was achieved by adding aliquots of the appropriate protein-free stock solution. Protein concentrations in aqueous solutions were determined based on UV absorption at 280 nm (DU 800 spectrophotometer, Beckman Coulter), using the HEWL extinction coefficient value of 2.64 g^−1^∙L∙cm^−1^ [51].

***Cloud Points:*** Cloud points were determined using a turbidity meter [52]. A freshly prepared aqueous protein sample (≈200 μL) was first incubated at 50 °C for ≈5 min prior to the turbidity experiment. At this relatively high temperature, which remains significantly lower than the HEWL unfolding temperature (≈75 °C) [53], our samples were homogeneous transparent mixtures above the solubility curve (see point (a) in Figure 2). The turbidity meter comprised a programmable circulating bath (1197P, VWR), a calibrated thermocouple (±0.1 °C), and a homemade optical cell, where the initially transparent sample (optical path of *L* = 0.4 cm) and a thermocouple probe were located [37]. Collimated light from a solid-state laser (633 nm, 5 mW, Coherent, Saxonburg, PA, USA) passed through the sample and its transmitted intensity, *I_T_*, was recorded by a photodiode detector coupled with a computer-interfaced optical meter (1835-C Newport, Irvine, CA, USA). After recording the transmitted intensity, *I_T_*_,0_, of the initially transparent sample, the temperature of the bath was lowered at a constant rate of −1 °C/min, and both temperature and transmitted intensity were continuously recorded. The temperature at which the turbidity, *τ* = (1/*L*)ln(*I_T_*_,0_/*I_T_*), showed a sharp increase upon cooling was chosen to identify the value of *T*_ph_ (cloud point). After achieving sample clouding via cooling (typically in the range from −15 to 5 °C), the temperature of the bath was steadily increased up to room temperature (≈22 °C). Note that HEWL undergoes cold denaturation below −10 °C only at very high pressures [54]. Cooling and heating profiles were combined to obtain temperature–turbidity hysteresis curves. Since cloud points are a function of the cooling rate, the LLPS temperature should be taken as the average between the cloud and clarification point [33]. Since this was not feasible for several samples due to the significant crystallization, cloud points at the rate of −1 °C/min were reported as the LLPS temperature in all cases.

***Light Microscopy:*** Cloudy samples were observed under a light microscope (Axioskop 40, Zeiss, Jena, Germany) at room temperature using bright-field and polarization microscopy. Images were taken using a digital camera (Axiocam MRc, Zeiss) interfaced by a computer with software (Axiovision AC 4.5, Zeiss).

***Determination of Crystallization Yield:*** A freshly prepared aqueous protein sample was prepared as described in Sample Preparation. Its protein concentration, *C_i_*, was spectrophotometrically determined. A known weighted amount of solution (≈100 μL) was placed in a test tube. Its volume, *V_i_*, was calculated from the sample mass using the known [55] specific HEWL volume of 0.713 cm^3^·g^−1^ and the density of the protein-free aqueous medium (measured using a DMA40 Mettler-Paar density meter), assuming volume additivity.

To determine the yield of protein crystallization, *Y*, a protein sample was first incubated at 50 °C for ≈30 min. The homogenous sample was then quenched at a temperature, *T*_Q_, near or below the LLPS temperature. The sample remained at this low temperature for the incubation time, Δ*t*_Q_. After time Δ*t*_Q_ is reached, the sample temperature was increased to 2 °C, well above the LLPS temperature, and remained at this temperature for 30 min, before sample characterization. The obtained sample suspension was quickly centrifuged at 2 °C using a centrifugal filter with a 0.2 μm pore size. All filtrates were found to be transparent through consistent visual inspection. This indicates that the transfer of crystalline materials through the filters is negligible. Concentration, *C_f_*, and volume, *V_f_*, of the filtered final homogeneous sample were then measured in the same way as described for the initial sample. Finally, crystallization yield, *Y*, was calculated using the following mass-conservation formula: *Y* = 1 − (*C_f_ V_f_*)/(*C_i_ V_i_*). The yield is typically determined a second time to assess the reproducibility of the data. Typically, variations in yield values of the order of 5% or less were obtained from samples quenched below the LLPS temperature, *T*_ph_. However, more significant variations were observed when crystallizations yields were measured when *T*_Q_ was appreciably above *T*_ph_. In these cases, multiple yield values were determined. 

## 4. Conclusions

It is known that NaCl is an efficient salting-out agent for HEWL. Indeed, it is the most employed crystallizing agent of this protein. It decreases the solubility of HEWL crystals [34,56] and induces a metastable LLPS in its aqueous solutions through cooling [21,23,31]. Interestingly, the addition of HEPES to NaCl significantly boosts HEWL crystallization, with the impact of HEPES becoming even greater below the LLPS temperature. Our measurements of HEWL crystallization yield, *Y*, below the LLPS temperature show that the combination of HEPES and NaCl produces a more than three-fold increase in *Y* compared to samples containing only NaCl at the same ionic strength of 0.2 M. Values of *Y* above 90%, close to the theoretical thermodynamic limit, can be reached within an operational time of the order of one hour with a high reproducibility. Measurements of crystallization yield from samples incubated below and above LLPS temperature show that LLPS significantly enhances protein crystallization. This is attributed to LLPS being able to promote crystal nucleation through wetting and two-step mechanisms.

The high and reproducible crystallization yield obtained from the LLPS-mediated crystallization of HEWL in the presence of HEPES at a fairly low ionic strength is especially remarkable for its relevance to protein purification protocols. However, it is important to recognize that HEWL is a model protein, known to have a high success rate in crystallization experiments. Thus, the success of LLPS-mediated crystallization for other proteins still needs to be experimentally demonstrated. Nonetheless, our results indicate a possible promising strategy for increasing the success of protein crystallization. This strategy consists of exploiting LLPS in the presence of two additives. The first additive is necessary to induce LLPS and establish the protein–protein attractive interactions necessary for protein crystallization. This is NaCl for HEWL, but it could be polyethylene glycol (PEG) [57,58,59,60] for other proteins. Indeed, PEG, together with ammonium sulfate, is regarded as the most successful crystallizing agent for proteins in general [11]. It is also extensively employed for inducing LLPS in protein aqueous solutions [30,52,61,62,63,64,65]. The second additive is a water-soluble organic molecule with multiple functional groups. This should be able to weakly bind to proteins in a solution and accumulate in the protein-rich liquid phase. Its protein-binding ability should be even more important inside a protein crystal, where it can stabilize crystal lattices by introducing cross-linking interactions between protein units [40]. From a thermodynamic point of view, the second additive should be able to increase the metastability gap [17,38,61] between the LLPS boundary and crystal solubility in the phase diagram (see Figure 1B). It is important to note that a library of this type of additives has already been proposed in the crystallography field, with systematic studies demonstrating that multi-functional organic molecules increase the success rate of protein crystallization [40]. However, a corresponding systematic study in which these additives are exploited in the context of LLPS-mediated crystallization is still missing. Other organic molecules that can bind to proteins could also be identified using either available protein–ligand crystal structures or molecular docking software.

In conclusion, we hope that our results on the yield of the LLPS-mediated crystallization of HEWL can lead to the development of crystallization protocols that are successful for other proteins, with applications in protein purification and the preparation of protein-based materials in general.

## Figures and Tables

**Table 1:** Ionic strengths (*I*), cloud points (*T*_ph_) and protein crystallization yields (*Y*) of various aqueous media at pH 7.4 containing HEWL at a concentration of 50 g·L^−1^.

Aqueous Media	*I*/M	*T*_ph_/°C	*Y*/%
NaCl (0.15 M) + HEPES (0.10 M)	0.20	−13.6	93 ^a,b^
NaCl (0.183 M) + Tris (0.020 M)	0.20	−6.8 ^c^	27
NaCl (0.10 M) + Na Phosphate (0.10 M)	0.32	−1.6	6
Na Phosphate (0.20 M)	0.44	−4.3	6
Na Phosphate (0.10 M) + HEPES (0.10 M)	0.27	−7.4	42

^a^ Yields extracted after the incubation of samples at −15 °C for 30 min below LLPS temperature, followed by incubation at 2 °C for 30 min above LLPS temperature. ^b^ Uncertainties for yields are ≈5%. ^c^ LLPS temperature extracted from Reference [37].

## Data Availability

Data are contained within the article.
